# Supplementary material for: Fatty Acid Binding Protein 5 Mediates Cell Death by Psychosine Exposure through Mitochondrial Macropores Formation in Oligodendrocytes
Source: Biomedicines. 2020 Dec 20;8(12):635. doi: 10.3390/biomedicines8120635 (PMC7766880; doi:10.3390/biomedicines8120635)
Supplement: Supplementary file 1 [file biomedicines-08-00635-s001.pdf]

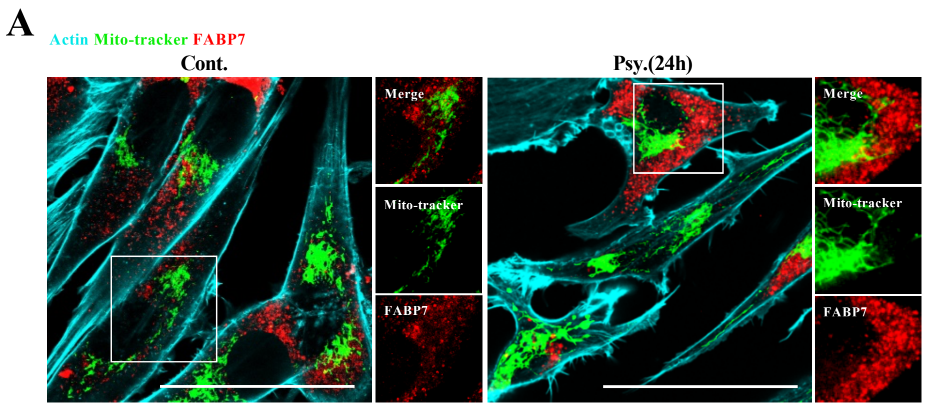

Figure S1: FABP7 does not localize in mitochondria, even when treated with psychosine. (A) Confocal microscopy of FABP7 (red), Mito-tracker (green), and actin (cyan), showed no co-localization of FABP7 with mitochondria. Scale bar=50μm.

Figure 1—figure supplement 1

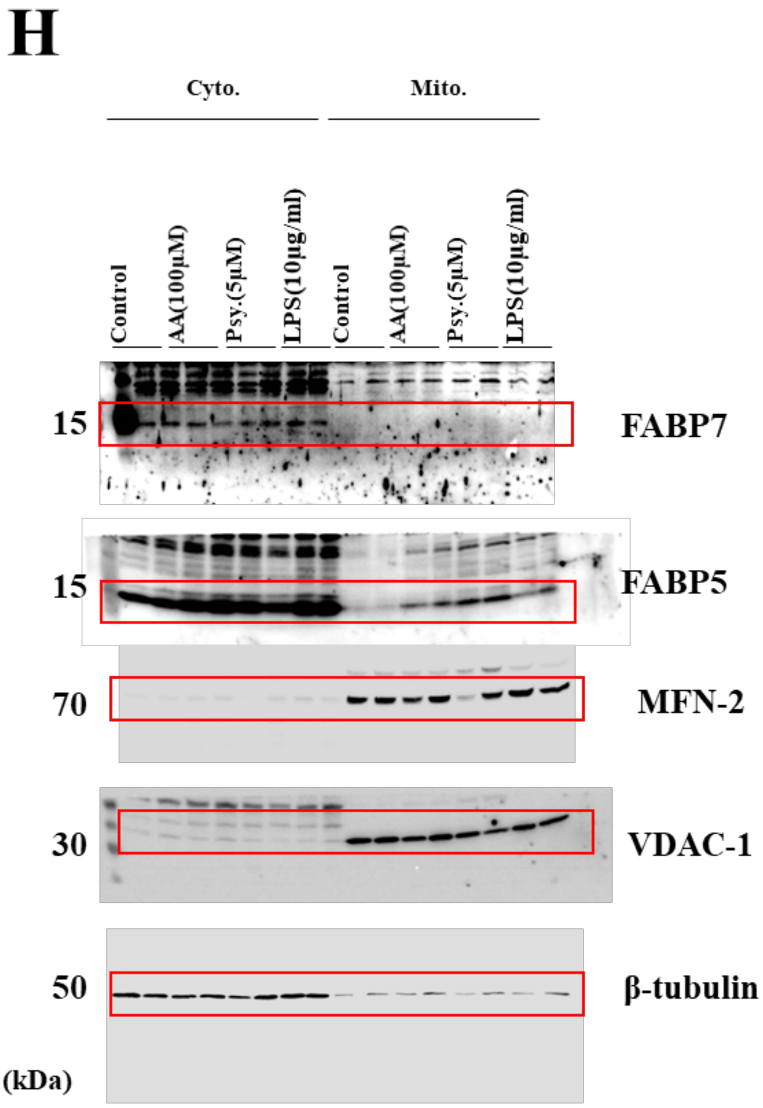

Figure 1— Original image 1

A

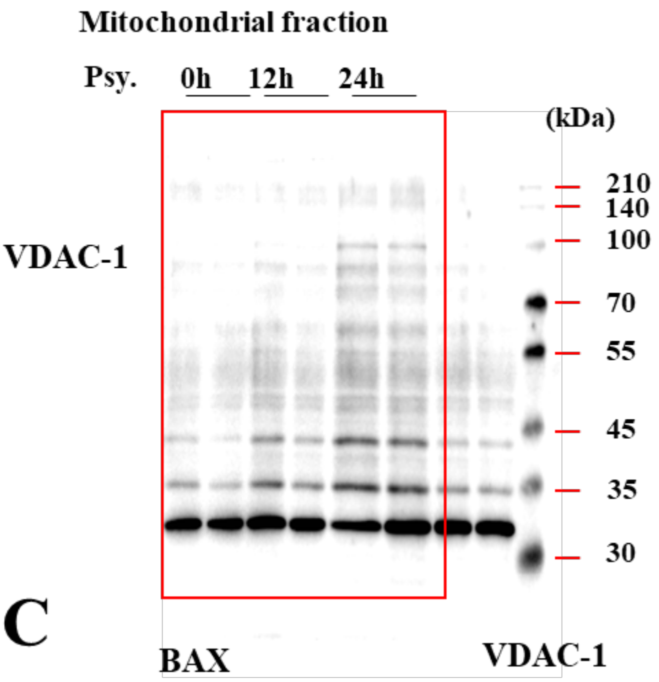

C

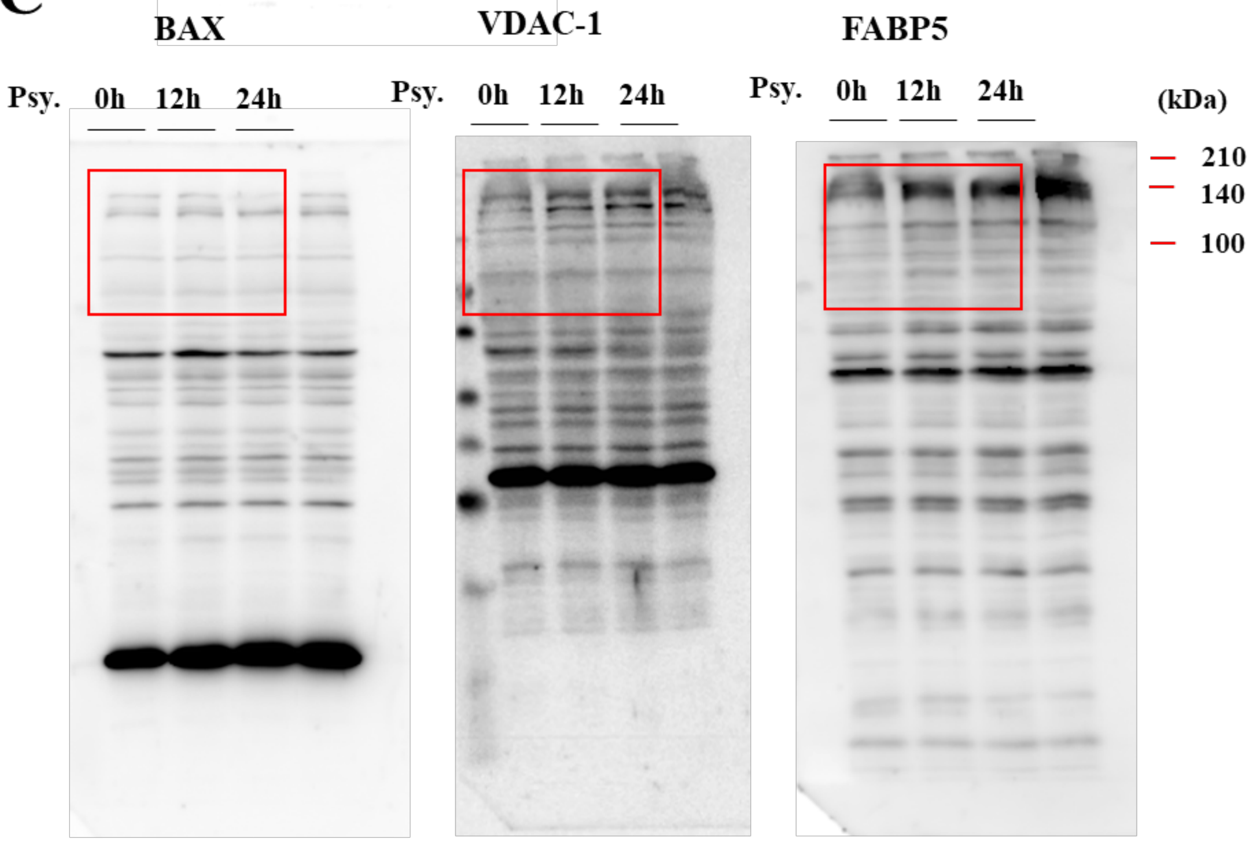

Figure 3—Original image 1

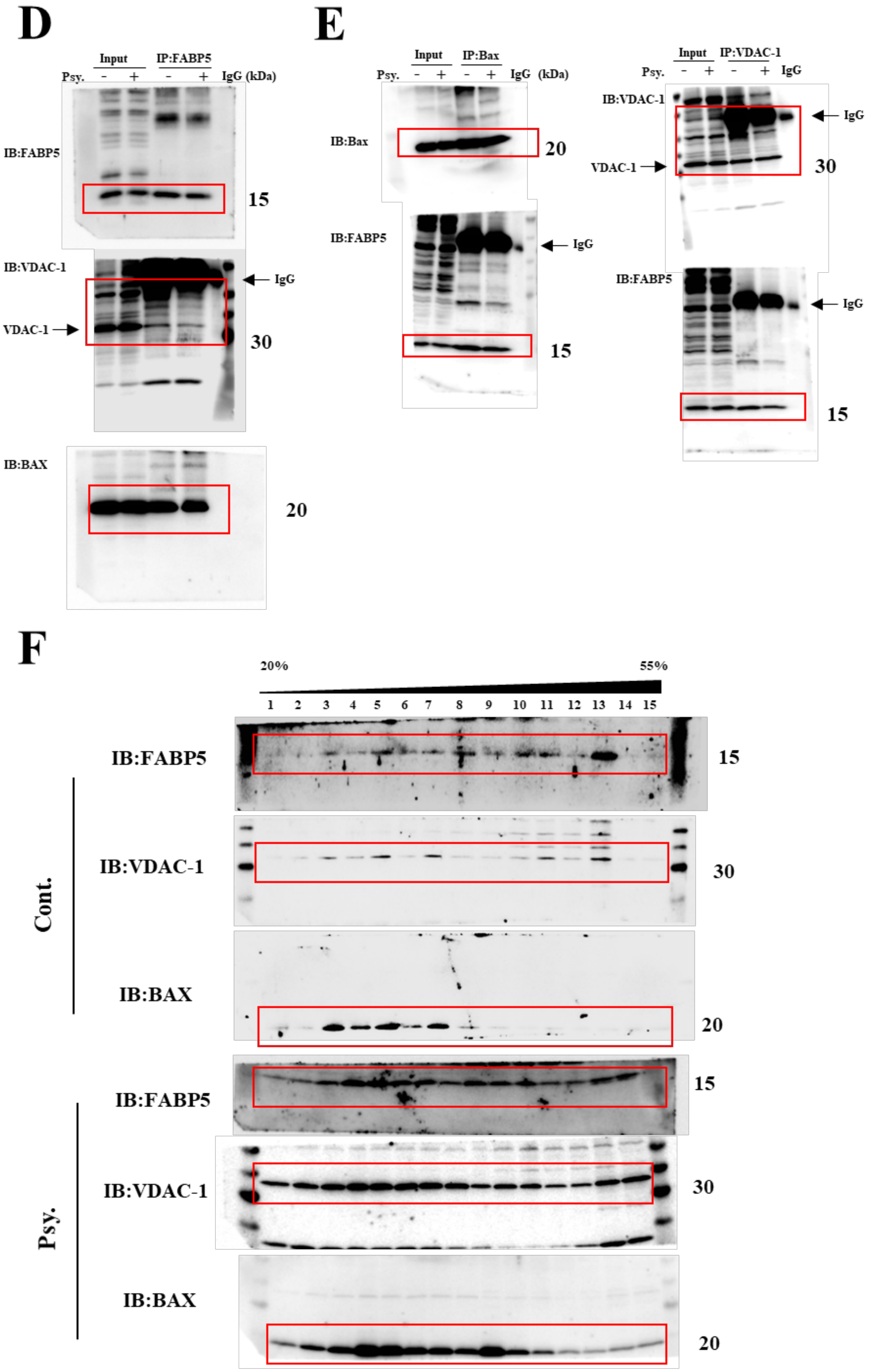

Figure 3—Original image 2

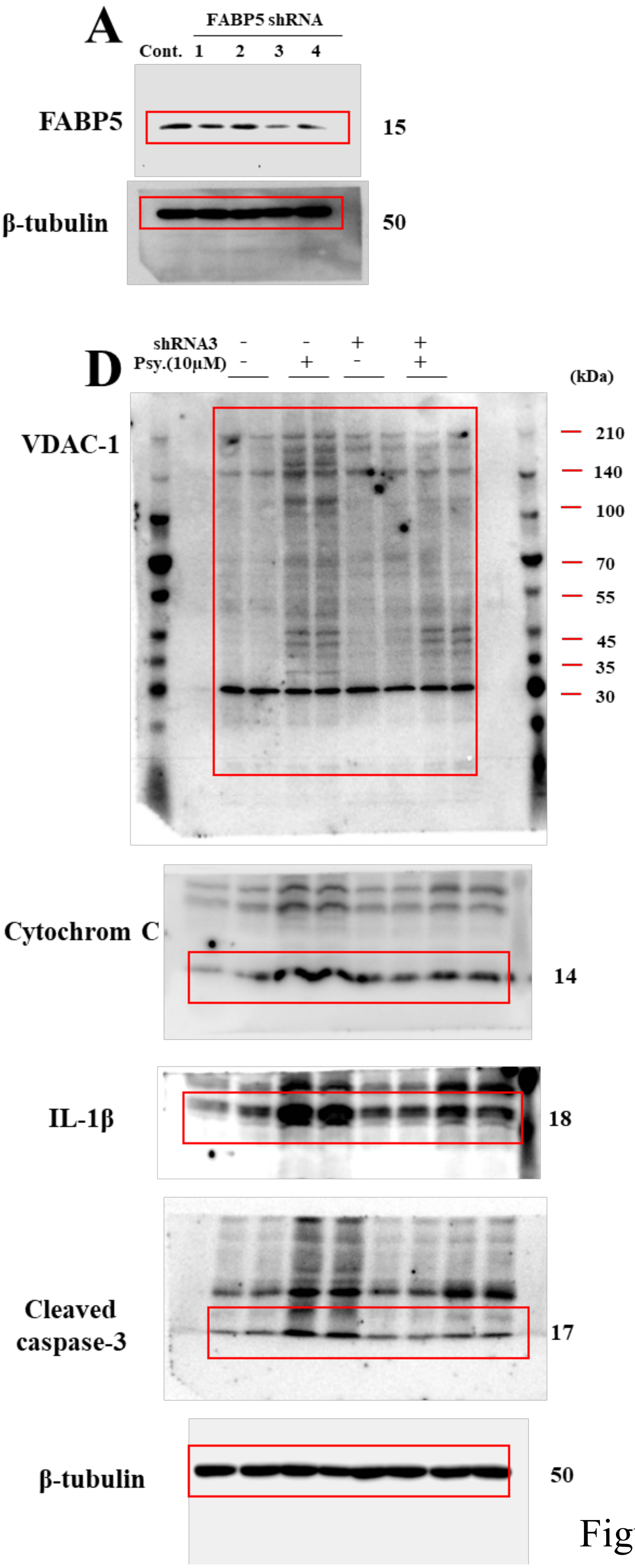

Figure 4—Original image

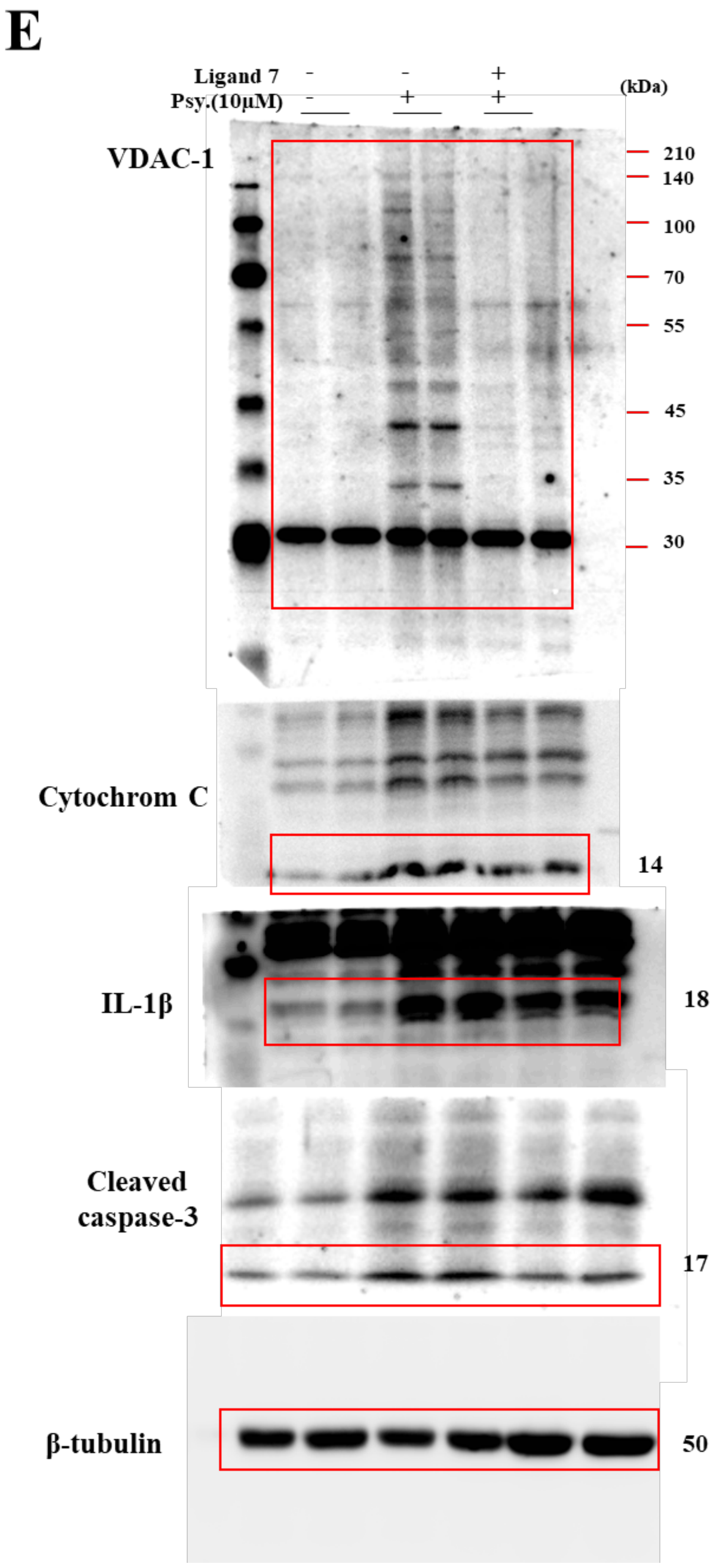

Figure 5—Original image
